# Characterizing User Engagement With a Digital Intervention for Pain Self-management Among Youth With Sickle Cell Disease and Their Caregivers: Subanalysis of a Randomized Controlled Trial

**DOI:** 10.2196/40096

**Published:** 2022-08-30

**Authors:** Chitra Lalloo, Fareha Nishat, William Zempsky, Nitya Bakshi, Sherif Badawy, Yeon Joo Ko, Carlton Dampier, Jennifer Stinson, Tonya M Palermo

**Affiliations:** 1 Department of Child Health Evaluative Sciences The Hospital for Sick Children Toronto, ON Canada; 2 Institute of Health Policy, Management and Evaluation University of Toronto Toronto, ON Canada; 3 Division of Pain & Palliative Medicine Connecticut Children's Medical Center Hartford, CT United States; 4 Division of Pediatric Hematology/Oncology/Blood and Marrow Transplant Department of Pediatrics Emory University School of Medicine Atlanta, GA United States; 5 Aflac Cancer and Blood Disorders Center Children's Healthcare of Atlanta Atlanta, GA United States; 6 Division of Hematology, Oncology and Stem Cell Transplantation Ann & Robert H. Lurie Children's Hospital of Chicago Chicago, IL United States; 7 Child Health Behavior & Development Seattle Children’s Hospital Seattle, WA United States; 8 Lawrence S. Bloomberg Faculty of Nursing University of Toronto Toronto, ON Canada

**Keywords:** engagement, adolescents, caregivers, sickle cell, pain, mHealth, self-management, digital health analytics, mixed methods, youth, management, disease, acute pain, chronic pain, coping, North America, intervention, child, digital health, program

## Abstract

**Background:**

Sickle cell disease (SCD) is characterized by severe acute pain episodes as well as risk for chronic pain. Digital delivery of SCD pain self-management support may enhance pain self-management skills and accessibility for youth. However, little is known about how youth with SCD and their caregivers engage with digital health programs. iCanCope with pain is a digital pain self-management platform adapted for youth with SCD and caregivers through a user-centered design approach. The program was delivered via a website (separate versions for youth and caregiver) and mobile app (youth only).

**Objective:**

We aimed to characterize patterns of user engagement with the iCanCope with SCD program among youth with SCD and their caregivers.

**Methods:**

A randomized controlled trial was completed across multiple North American SCD clinics. Eligible youth were aged 12-18 years, diagnosed with SCD, English-speaking, and experiencing moderate-to-severe pain interference. Eligible caregivers were English-speaking with a child enrolled in the study. Dyads were randomized to receive the iCanCope intervention or attention-control education for 8-12 weeks. This report focused on engagement among dyads who received the intervention. User-level analytics were captured. Individual interviews were conducted with 20% of dyads. Descriptive statistics characterized quantitative engagement. Content analysis summarized qualitative interview data. Exploratory analysis tested the hypothesis that caregiver engagement would be positively associated with child engagement.

**Results:**

The cohort included primarily female (60% [34/57] of youth; 91% [49/56] of caregivers) and Black (>90% of youth [53/57] and caregivers [50/56]) participants. Among 56 dyads given program access, differential usage patterns were observed: both the youth and caregiver engaged (16/56, 29%), only the youth engaged (24/56, 43%), only the caregiver engaged (1/56, 2%), and neither individual engaged (16/56, 29%). While most youth engaged with the program (40/57, 70%), most caregivers did not (39/56, 70%). Youth were more likely to engage with the app than the website (85% [34/57] versus 68% [23/57]), and the most popular content categories were goal setting, program introduction, and symptom history. Among caregivers, program introduction, behavioral plans, and goal setting were the most popular content areas. As hypothesized, there was a moderate positive association between caregiver and child engagement (χ^2^_1_=6.6; *P*=.01; ϕ=0.34). Interviews revealed that most dyads would continue to use the program (11/12, 92%) and recommend it to others (10/12, 83%). The reasons for app versus website preference among youth were ease of use, acceptable time commitment, and interactivity. Barriers to caregiver engagement included high time burden and limited perceived relevance of content.

**Conclusions:**

This is one of the first studies to apply digital health analytics to characterize patterns of engagement with SCD self-management among youth and caregivers. The findings will be used to optimize the iCanCope with SCD program prior to release.

**Trial Registration:**

ClinicalTrials.gov NCT03201874; https://clinicaltrials.gov/ct2/show/NCT03201874

## Introduction

Sickle cell disease (SCD) is the most common genetic blood condition in North America and primarily affects people of African descent [1]. The hallmark feature of SCD is recurrent episodes of acute severe pain due to vaso-occlusive crisis [2]. Vaso-occlusive crisis occurs when sickled red blood cells block blood flow, resulting in tissues becoming deprived of oxygen and causing pain. SCD pain is reportedly worse than postoperative pain and as intense as terminal cancer pain, and it can negatively impact all aspects of health-related quality of life [3-5]. Youth tend to experience increased SCD pain burden as they age into adolescence and young adulthood, with increased health care utilization [4-7]. In addition to acute pain from vaso-occlusive crisis, many youth with SCD also experience daily chronic pain [8,9]. The negative consequences of acute and chronic SCD pain can include depression and anxiety, academic underachievement related to missing school, limited opportunities for social interaction with peers, impaired physical activity, poor sleep, and high stress [9]. The vast majority of SCD pain episodes are treated in the home setting [10]. Unfortunately, many of these episodes may not be optimally managed with pharmacological approaches alone [11-13].

Self-management has been defined as “the individual’s ability to manage the symptoms, treatment, physical and psychological consequences, and lifestyle changes inherent to living with a chronic illness” [14]. The most successful self-management interventions are rooted in the principles of cognitive behavioral therapy [15-18]. Cognitive behavioral therapy involves normalization of the patient’s experience through education, training in strategies for managing disease-related symptoms and other stressors, enhancing self-efficacy, and guidance on developing and maintaining a long-term self-management plan. Gaining skills in monitoring and managing SCD symptoms independently is particularly critical to achieve early in the disease trajectory, as many youth experience worsened pain and disability in adulthood [19].

Barriers to implementing cognitive behavioral therapy in SCD populations include the challenge of delivering pain self-management interventions in traditional office-based visits, costly resources required to provide such support, and maintaining patient engagement [12,20]. Ubiquitous consumer technologies (eg, smartphones) offer opportunities to enhance the accessibility and interactivity of self-management support for youth with SCD [21,22].

iCanCope with pain is a digital pain self-management platform originally developed for youth with chronic pain [23-25]. The platform was adapted for youth with SCD through a user-centered design approach, including a qualitative needs assessment and design sessions [12,26]. The iCanCope with SCD program is currently undergoing efficacy evaluation through a multi-centered randomized clinical trial (NCT03201874) [26]. In the present report, we explore engagement with the digital intervention during the course of this trial.

Little is known about how youth with SCD and their caregivers engage with digital health interventions, and research to date has focused mostly on internet-delivered programs [27]. A scoping review focusing on user engagement with mobile health apps did not identify any studies specific to SCD [28]. Moreover, there is little standardization of engagement metrics due to the heterogeneity of available digital health programs. To add to this body of knowledge, our goal was to operationally define user engagement (tied to program content) with the iCanCope with SCD pain self-management program, to characterize patterns of user engagement among youth with SCD and their caregivers, and to identify individual youth and caregiver perspectives about the digital intervention. Lastly, in an exploratory fashion, we tested the *a priori* hypothesis that caregiver engagement would be positively associated with child engagement.

## Methods

### Study Design

A 2-arm randomized trial design was used. Dyads were randomized to 1 of 2 possible study conditions: (1) iCanCope with SCD intervention or (2) attention-control education. Details on the trial methodology are available in an open-access protocol paper [26], and the trial has been registered on ClinicalTrials.gov (NCT03201874). This paper will focus on program engagement data from dyads who received the intervention condition. Comparative efficacy results from the broader trial will be reported separately in a future paper.

### Ethics Approval

The study was approved by the locally responsible institutional ethics boards (Seattle Children’s Institutional Review Board, STUDY00001578; Emory University Institutional Review Board, 00092216; Connecticut Children’s Institutional Review Board, 17-115-CCMC; and The Hospital for Sick Children Research Ethics Board, 1000053724).

### Participants

The main recruitment sites were Connecticut Children’s Medical Center, Children’s Healthcare of Atlanta/Emory University, Seattle Children’s Hospital, and The Hospital for Sick Children in Toronto. In addition, study referrals were accepted from University of Mississippi Medical Center, Boston Medical Center, and Northwestern University/Lurie Children’s Hospital. The study was centrally managed at Seattle Children’s Research Institute.

Youth were eligible if they (1) were aged between 12 and 18 years, (2) were diagnosed with any SCD genotype, (3) were able to speak and read English, (4) scored at least 4 (indicating moderate-to-severe pain interference over the past month) on the Sickle Cell Pain Burden Interview [6], and (5) were willing and able to complete online measures. Youth were excluded if they had significant cognitive limitations that would impair their ability to use and understand the iCanCope program, as per their health care provider or caregiver. Youth were also excluded if they had received more than 4 sessions of outpatient psychological therapy for pain management in the 6 months prior to the time of screening.

Caregivers were eligible if (1) they were able to speak and read English, (2) they were willing and able to complete online measures, and (3) their child was a study participant. Youth were permitted to enroll in the study even if their caregiver chose not to participate.

### Procedure

Dyads assigned to the intervention condition were given user-authenticated access to the iCanCope with SCD program for a period of 8-12 weeks. The program content, based on the principles of cognitive behavioral therapy, was delivered via a modular website and complementary smartphone app as summarized in Tables 1 and 2. Standardized orientation materials were used to guide participants on how to login and independently use the program. Separate websites with unique content were provided for youth and caregiver participants, while a smartphone app (iOS/Android) was provided for youth participants only. Website navigation can be found in Figures S1 and S2 in Multimedia Appendix 1. The youth and caregiver websites were each organized into 6 core modules in addition to 2 optional youth modules on insomnia and negative mood. Features of the smartphone app included a daily symptom tracker (ie, pain intensity, pain impact, mood, and sleep quality), a calendar to view historical symptom data, goal setting in relevant domains (eg, sleep and mood), a library of SCD education and pain coping strategies, and a community forum to interact with other users (Figure S3 in Multimedia Appendix 1).

**Table 1:** iCanCope with sickle cell disease program content and delivery for youth.

iCanCope with SCD^a^ program		Content delivery	
Content category	Example content	Website	App
Introduction to the program	General information about the iCanCope with SCD program and what to expect	Yes	Yes
About pain management	3Ps of pain management (psychological, physical, and pharmacological strategies)	Yes	Yes
About SCD and treatment	Genetics, potential impact of SCD on daily life, and treatment options	Yes	Yes
Goal setting	Guidance on how to formulate specific, measurable, achievable, relevant, and timebound goals; a feature within the smartphone app where users could set and track personalized goals to improve activity, mood, and sleep	Yes	Yes
Symptom tracking	A feature within the smartphone app where users could report daily pain intensity, pain impact, mood, and sleep quality and view their symptom trends	N/A^b^	Yes
History	A feature within the smartphone app where users could view previously reported symptoms	N/A	Yes
Community support	A forum feature within the smartphone app where users could post responses to community questions and “favorite” the posts of other app users	N/A	Yes
Stress, relaxation, and negative emotions	Reducing negative thoughts, thought stopping, deep breathing exercises, muscle relaxation, imagery, scheduling pleasant activities, and finding the positives	Yes	Yes
Sleep and insomnia	Pain and sleep, healthy sleep habits, ways to fall and stay asleep, and how to think differently about sleep	Yes	Yes
Communication and self-advocacy	Communication skills, talking with the health care team, and talking with the school	Yes	Yes
Healthy lifestyle and looking ahead	Pacing, graded activity, hydration, considerations for the future, and transition to adult health care	Yes	Yes

^a^SCD: sickle cell disease.

^b^N/A: not applicable.

**Table 2:** iCanCope with sickle cell disease program content and delivery for caregivers.

iCanCope with SCD^a^ program		Content delivery	
Content category	Example content	Website	App
Introduction to the program	What teens are learning in the program; specific, measurable, achievable, relevant, and timebound goals to support teens	Yes	N/A^b^
Behavioral plans	How to create behavioral plans to increase teen adaptive behaviors	Yes	N/A
Problem solving	Learn how to approach problems positively, identify problems effectively, and generate and implement solutions	Yes	N/A
Communication	Strategies to help communicate with teens, health care providers, and school staff	Yes	N/A
Wrap-up	Review of key concepts	Yes	N/A

^a^SCD: sickle cell disease.

^b^N/A: not applicable.

### Outcome Measurement

#### Baseline Characteristics

At baseline, caregivers completed a background questionnaire to capture sociodemographic data (eg, age, race, and ethnicity), and both caregivers and youth completed information about their technology access and usage. Youth were screened into the study using the Sickle Cell Pain Burden Interview to assess SCD pain burden in the past month. The Sickle Cell Pain Burden Interview yielded a total score ranging from 0 (no pain burden) to 28 (severe pain burden). Dyads additionally completed several other clinical effectiveness outcomes detailed in an open-access protocol paper [26].

#### Primary Outcome

##### Quantitative

User-level analytics were captured for the app and website components of the iCanCope with SCD program. App engagement was characterized using APEEE (Analytics Platform to Evaluate Effective Engagement) [29], while website engagement data were captured using Google Analytics.

##### Qualitative

To gather perceptions of the treatment program from youth and caregivers, individual semistructured interviews were conducted with a convenience subset of dyads representing approximately 20% of the intervention group. All dyads were invited to complete an interview following their posttreatment assessment and prior to their 6-month follow-up assessment. Recruitment for the interview continued until 12 caregivers and youth completed the interviews. Separate interviews were conducted with youth and caregivers. The interview guide was designed to capture perspectives on the experience of using the iCanCope with SCD program, including likes, dislikes, value, and areas for improvement. Interviews were 15-20 minutes in duration, conducted over the telephone, and audio recorded for later transcription.

### Data Analysis

Quantitative data were analyzed using STATA version 15.1 (Stata Statistical Software). Descriptive statistics were used to summarize background characteristics of the sample and program engagement data. An engagement interaction with the website was defined as a unique content page view. For the app, the following engagement interactions were captured: symptom check-in completed; goal created; library article viewed; and interaction within the community forum. For the youth-specific intervention, program content was distributed across the website and app such that an individual user might access topic-specific information (eg, “healthy sleep habits”) by viewing a website page or reading an app library article. Given this overlap in program content delivery, engagement data for youth participants were also mapped by content category (Table 1).

To address our exploratory aim regarding the relationship between parent engagement and child engagement, we conducted a chi-square test (significance was set at α<.05) and assessed the strength of the association using the phi correlation. The phi correlation can range from 0 to 1 and was interpreted as follows: no correlation or very weak correlation (0-0.19), weak correlation (0.20-0.29), moderate correlation (0.30-0.49), strong correlation (0.50-0.69), and very strong correlation (0.70-1.00) [30].

Audio recordings from the qualitative interviews were transcribed verbatim and analyzed by 2 team members (CL and FN) using Dedoose Version 9.0.17 (SocioCultural Research Consultants, LLC). Simple content analysis, a dynamic process that summarizes the informational content of data, was used [31,32]. Specifically, data for all participants were coded and organized into categories that reflected the emerging themes. The raw data were revisited on a regular basis throughout the analytic process to ensure that the codes were grounded in the data. Any disagreements were resolved through consensus.

Participant recruitment spanned periods before and after onset of the COVID-19 pandemic. For analysis purposes, March 2020 was used to differentiate between *prepandemic* and *pandemic* recruitment periods in North America.

## Results

### Baseline Characteristics

Recruitment was carried out between January 1, 2018, and September 30, 2021. A total of 57 youth and 56 caregivers (56 dyads and 1 youth-only participant) were randomized to the iCanCope with SCD intervention condition and received instructions on how to access the program. The sample included dyads who enrolled in the study before (43/56, 75%) and after (14/56, 25%) pandemic onset. Demographic characteristics of the sample are provided in Tables 3 and 4.

Nearly all youth participants (55/57, 96%) were in middle school or high school. Most youth (47/57, 82%) reported using a smartphone multiple times per day. Caregiver participants (n=56) reported their highest completed education level as high school or less (11/56, 20%), vocational or trade school or some college/university (15/56, 27%), college or university (18/56, 32%), or graduate degree or professional school (11/56, 20%). Most dyad households (30/56, 54%) included 1 or 2 children under 18 years of age (range of 1 to 6 children). With regard to the total annual household income before taxes, participants reported less than US $24,999 (11/56, 20%), US $25,000-49,999 (16/56, 29%), US $50,000-74,999 (9/56, 16%), US $75,000-99,999 (9/56, 16%), and US $100,000 or more (5/56, 9%).

**Table 3:** Characteristics of youth participants.

Characteristic		Youth value (N=57)
Age (years), mean (SD)		14.8 (2.0)
**Sex, n (%)**		
	Female	34 (60)
	Male	23 (40)
**Gender identity^a^, n (%)**		
	Female	34 (60)
	Male	23 (40)
**Race, n (%)**		
	American Indian	1 (2)
	Black	53 (93)
	Latino/Hispanic	1 (2)
	Asian	2 (4)
**Ethnicity^b^, n (%)**		
	Hispanic	5 (9)
	Non-Hispanic	46 (84)
	Unknown	4 (7)
**SCD^c^ genotype^d^, n (%)**		
	Hemoglobin SS	35 (66)
	Hemoglobin SC	12 (23)
	Hemoglobin S beta thal plus	3 (6)
	Hemoglobin S beta thal zero	2 (4)
	Unknown	1 (2)
SCD Pain Burden Interview score, mean (SD)		10.9 (5.8)
Currently taking hydroxyurea, n (%)		40 (70)
Receiving regular blood transfusions, n (%)		8 (14)

^a^Response options were available for additional gender identities including transgender.

^b^Missing for ethnicity (n=2).

^c^SCD: sickle cell disease.

^d^Missing for SCD genotype (n=4).

**Table 4:** Characteristics of caregiver participants.

Characteristic			Caregiver value (N=56)	
**Relationship with the youth participant^a^, n (%)**				
	Biological mother	49 (89)		
	Biological father	3 (5)		
	Brother	2 (4)		
	Step-mother	1 (2)		
**Marital status^b^, n (%)**				
	Common law or married	23 (44)		
	Separated or widowed	8 (15)		
	Single	21 (40)		
**Race, n (%)**				
	Black	51 (90)		
	White	1 (2)		
	Other^c^	2 (4)		
	Mixed	2 (4)		
**Ethnicity^d^, n (%)**				
	Hispanic	3 (5)		
	Non-Hispanic	48 (87)		
	Unknown	4 (7)		

^a^Missing for relationship to youth participant (n=1).

^b^Missing for marital status (n=4).

^c^Reported races were Dominican and Greek.

^d^Missing for ethnicity (n=1).

### Quantitative Results

#### Dyad-Level Program Engagement

Of the 56 dyads who were given access to the iCanCope with SCD program, differential usage patterns were found. Engagement by both the youth and caregiver occurred for 29% (16/56) of the dyads, whereas it was more likely for only youth to engage in the program (24/56, 43%). In 1 dyad out of 56 (2%), only the caregiver engaged with the intervention, and neither individual engaged for 29% (16/56) of dyads. The 1 youth participant who joined the study without a corresponding caregiver did not engage with the program.

**Figure 1:**
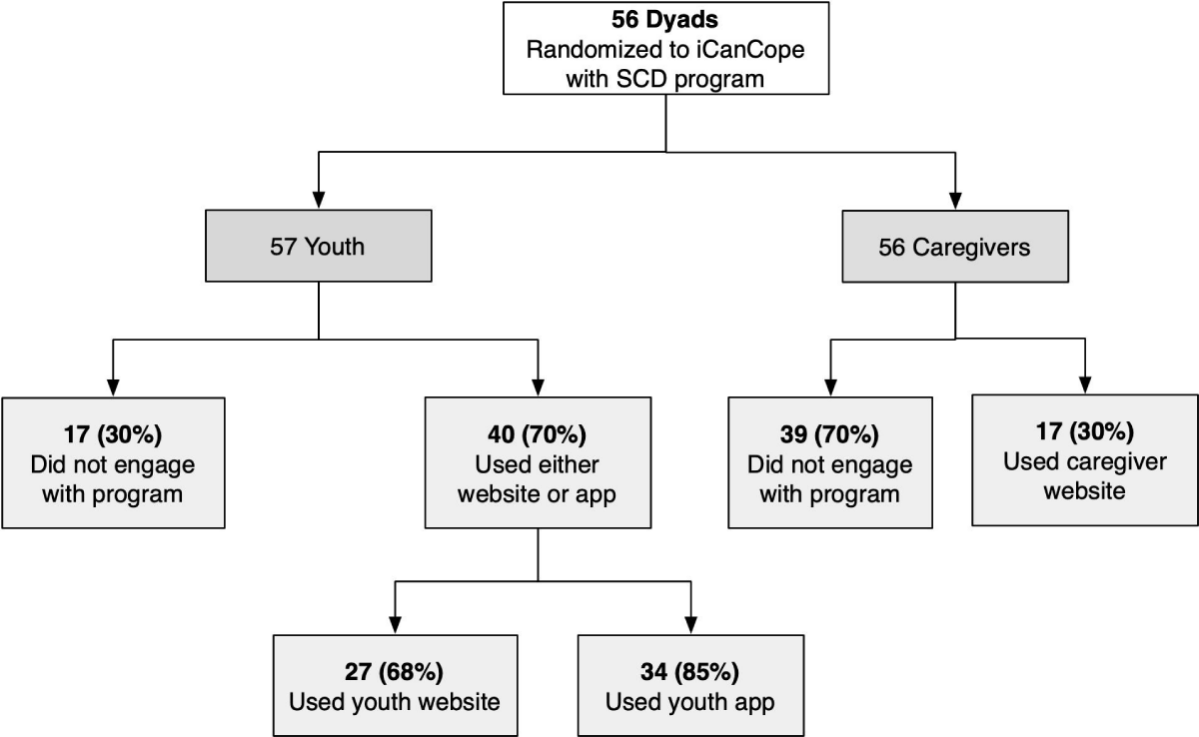
Engagement of youth and caregivers with components of the iCanCope with sickle cell disease (SCD) intervention.

**Figure 2:**
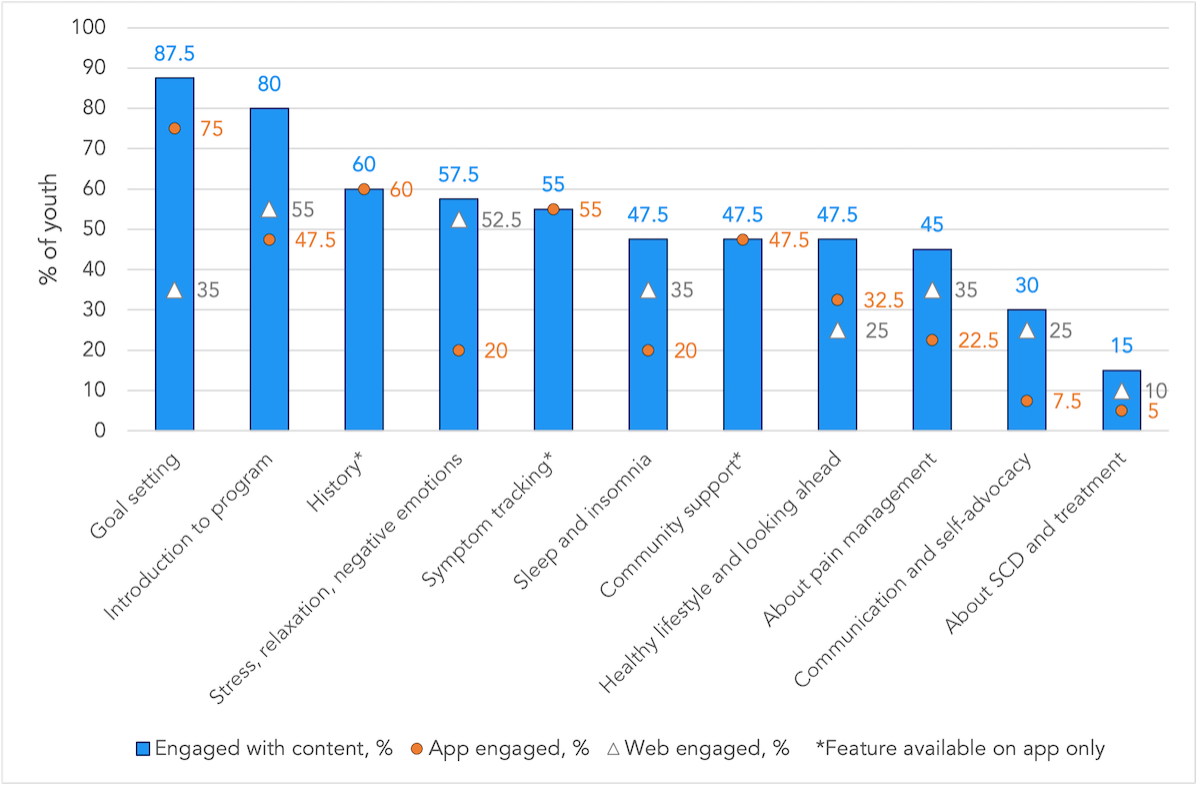
Differential content engagement among youth who used the iCanCope with sickle cell disease program (N=40). SCD: sickle cell disease.

**Figure 3:**
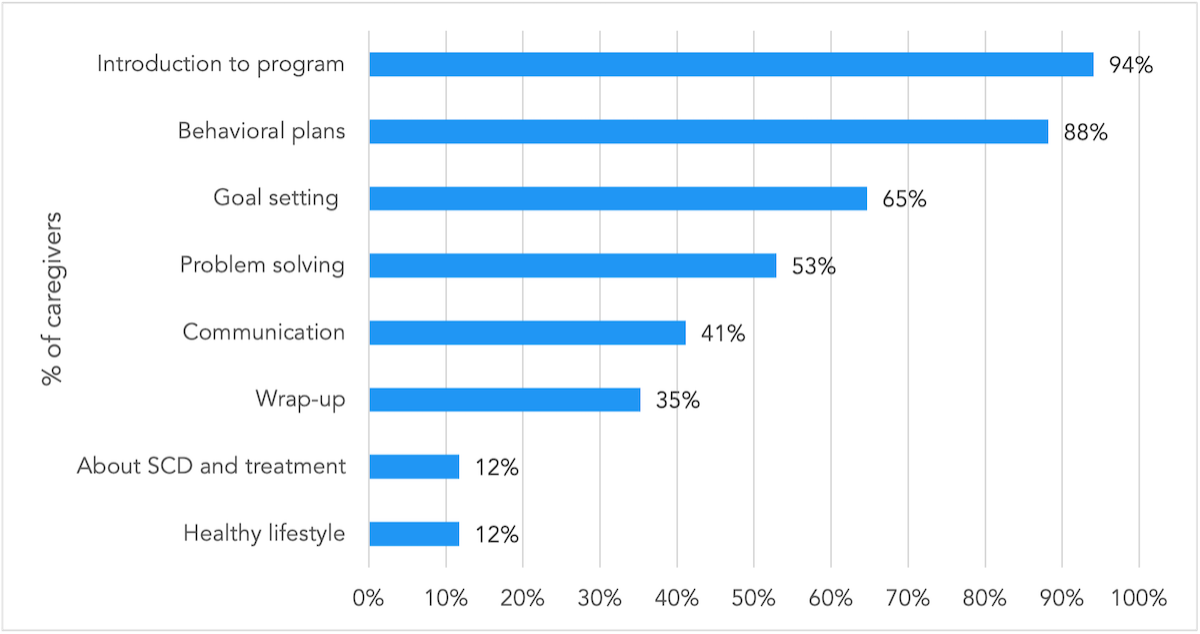
Differential content engagement among caregivers who used the iCanCope with sickle cell disease program (N=17). SCD: sickle cell disease.

Differential engagement of youth and caregivers with components of the iCanCope with SCD intervention is visualized in Figure 1. Content-specific engagement among youth and caregivers is summarized in Figure 2 and Figure 3, respectively. Among the content categories, the most popular for youth were goal setting, introduction to the program, and history. For caregivers, the most popular content categories were introduction to the program, behavioral plans, and goal setting.

#### Exploratory Predictor of Treatment Engagement

As hypothesized, there was a moderate positive association between caregiver engagement and child engagement with the intervention (χ^2^_1_=6.6; *P*=.01; ϕ=0.34).

### Qualitative Results

#### Interview Participants

Individual interviews were completed with 12 dyads between September 2019 and October 2021, representing participants who completed the study either before (5/12, 42%) or after (7/12, 58%) onset of the COVID-19 pandemic. Youth interviewees ranged in age from 12 to 18 years, and most were female (10/12, 83%). All interviewed caregivers were mothers. The interview sample included dyads where both the youth and caregiver engaged with the program (5/12, 42%), only the youth engaged (6/12, 50%), and only the caregiver engaged (1/12, 8%). Among youth interviewees, 11 of 12 (92%) used either the website or app, 7 of 12 (58%) used the website, and 10 of 12 (83%) used the app. Among caregiver interviewees, 6 of 12 (50%) viewed the website content during the study period and the remainder did not engage with the program.

#### Motivation for Program Use Among Youth

Nearly all youth interviewees (11/12, 92%) indicated that they intended to continue using the iCanCope with SCD program in the future. Moreover, youth who used the app were asked how they would hypothetically rate it on the iOS or Android mobile store between 0 and 5 stars. Among the 10 youth who used the app, 6 (60%) gave a rating of 5 stars and 4 (40%) gave a rating of 4 stars. As rationale for their ratings, 1 participant shared,

…I think this is the first app I’ve heard about sickle cell and I think it’s really informative and I like it.

Youth #126

**Figure 4:**
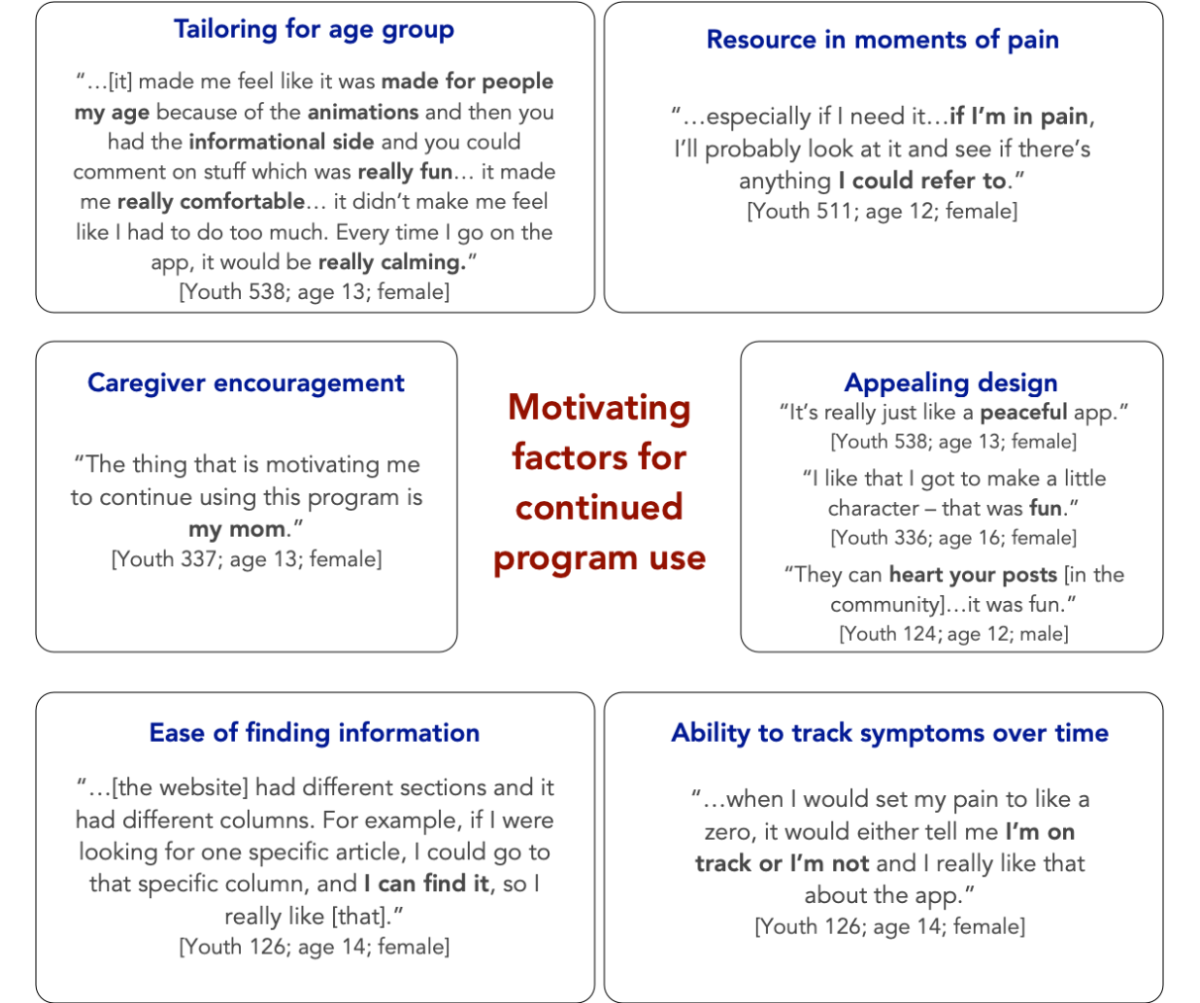
Motivation for continued use of the iCanCope with sickle cell disease program among youth interviewees.

Cited motivating factors for wishing to use the program after study completion are summarized in Figure 4.

#### Rationale for Program Recommendation Among Caregivers

Most caregiver interviewees (10/12, 83%) indicated that they would recommend the program to a friend or family member living with SCD. One caregiver shared,

I have a host of family members that deal with sickle cell, so I would also like to be able to visit the website and provide them with different coping mechanisms or different kind of information that I have found useful.

Caregiver #337

Acknowledging the ups and downs of SCD, another caregiver stated,

…let me help you cope with this craziness that’s going on in your life every day.

Caregiver #336

Several caregivers noted that they would be more likely to recommend the program to youth who are newly diagnosed with SCD or have frequent pain crises. For instance, 1 caregiver commented as follows:

…for someone that doesn’t have a lot of crises, that wouldn’t be helpful...for someone that [has] a lot of crises, this would be very helpful.

Caregiver #537

Similarly, a caregiver who characterized the program as a good refresher made the following statement:

I like the way it’s laid out in terms of the content being very layman terms. You know, there’s not a lot of – you’re not overwhelmed with a lot of medical things that people are saying ‘oh, well what’s this, and what’s that, and what’s this?’. So, in terms of that content, I think it’s fantastic. So, I would recommend it.

Caregiver #119

#### Exploring Reasons for Low Engagement

Among the 6 of 12 (50%) caregiver interviewees who did not use the program during the study period, the reasons for low engagement included a perceived lack of relevance to their circumstances.

…I found that some of the questions on it, things they would ask me - the exercises the [program] would ask me to do was sort of not relevant to me…I felt like it didn’t really apply to [my child] or to me.

Caregiver #127

This caregiver also noted that another reason for lack of engagement was the large amount of content.

…there’s so many sections within one unit itself. So, when I thought I was almost done, I still had more to do. I’m able to do only one section at a time or not even able to finish the whole unit because of timing.

…when I get home, I’m exhausted. I’m a nurse and I work in ICU so we’re very busy at work especially with the COVID…I live really far from work, so I leave home early, I get home really late, and it’s just busy that’s all but nothing else.

Among caregivers who did not use the program themselves, some noted that it was helpful for their children.

I think it made her more aware and more focused and…process…how she can cope with it and mostly like she’s not alone in this… that there are people out there that care. I think it really helped.

Caregiver #126

Others noted that the program helped them understand how to better support their children.

…it actually shed a lot of light into a couple of things especially when it gets to her mood swings and why she does certain things and also helping her manage her pain.

Caregiver #112

One youth interviewee who did not engage with the program did not provide a specific reason why but did indicate interest in using it after the study was complete.

…because I feel like it’s a good…way to…learn more about it and I could learn and study more myself.

Youth #520

#### Differential Engagement With Program Components

As shown in Figure 1, youth who engaged with the program were more likely to use the app (34/40, 85%) compared with the website (27/40, 68%). Reasons for this preference described by interviewees included perceived ease of use and acceptable time commitment.

I liked the app more because it was easier to use than the website…the app was simple, and it only took a couple of minutes.

Youth #537

The interactivity of the app feature set was also cited as a reason for use.

I like the app more than I like the website because of the daily diaries and connecting to others…and there [is] a library inside the app where you can read and how you can set goals.

Youth #126

This user continued with the following statement:

I think I have some downsides with the website because the website you only have to read, but the app – it’s fun.

While technical bugs were rare and minimally disruptive (eg, broken external resource link within the youth website and temporary app diary error), technical limitations of the website caused frustration among some participants and may have impacted engagement with that program component. For instance, a caregiver who had low program engagement noted the following:

…the website can’t save what you write, so every time you come back it does get deleted.

Caregiver #537

#### Promotion of Disease Self-management Behaviors

**Figure 5:**
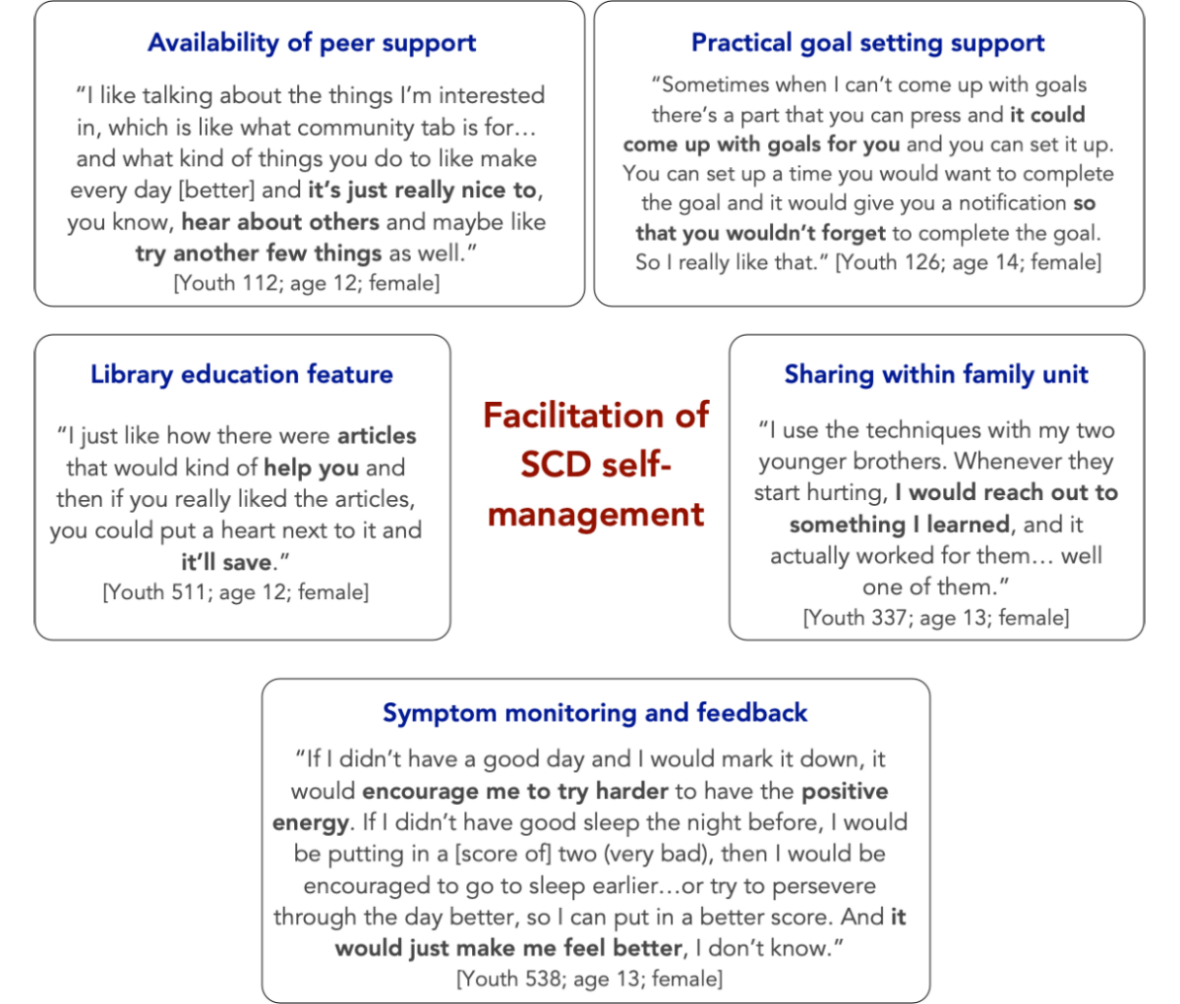
Examples of how the program facilitated disease self-management. SCD: sickle cell disease.

When asked about how they used the program in their day-to-day life, youth interviewees described several concrete ways that the program supported them to engage in SCD self-management, as summarized in Figure 5.

#### Influence of the Pandemic on Self-management

Youth who completed the study during the pandemic described that the program helped to mitigate some disruptions to their self-management routines.

I found the app very helpful because for me it can be very hard especially because I’m in high school…now that we’re online…I like do my work and…I just stay in my room and I don’t do the stuff that I’m supposed to do to ensure that I’m preventing crises. So, having the app, especially the goal chapter, was very helpful because I was able to put what goals I wanted. For example, if I wanted to drink more water or I wanted to get more sleep that week then I was able to do that. That motivated me to want to achieve the goal.

Youth #127

## Discussion

### Principal Findings

This is one of the first studies to characterize patterns of engagement with a web- and app-based pain self-management program among youth with SCD and their caregivers. The data demonstrate that most youth with SCD engaged with the program (40/57, 70%) while most caregivers did not (39/56, 70%). The youth program was delivered via a mobile app and a website. Youth were more likely to engage with the app than the website, and the most popular content categories were goal setting, introduction to the program, and symptom history. Among caregivers who engaged with the website, introduction to the program, behavioral plans, and goal setting were the most popular content areas. Exploratory analysis revealed a significant moderate association between caregiver and youth engagement with the intervention. The key insights from qualitative interviews were as follows: (1) nearly all youth intended to continue using the program; (2) caregivers would recommend the program to others living with SCD who were newly diagnosed or who frequently experienced pain crises; (3) caregivers cited the large amount of content and low relevance to their specific circumstance as reasons for low engagement; (4) youth preferred the app over the website because of perceived greater ease of use, acceptable time commitment, and interactivity; and (5) use of the youth program helped to facilitate SCD self-management behaviors such as goal setting, applying strategies for pain management, symptom trend monitoring, and learning through peer support.

### Comparison With Prior Work

There are several app- and web-based interventions designed for children and youth with SCD [33-36] that focus on enhancing SCD knowledge, medication adherence, quality of life, transition to adult care, and pain management. However, engagement or adherence metrics for these interventions were reported in only a few studies. Among 4 recent studies [34-36] that reported some engagement data, operational definitions varied, making direct comparisons difficult. First, Palermo et al reported high engagement with web-based management of adolescent pain (WebMAP), an internet-delivered cognitive-behavioral therapy intervention tailored for SCD [27]. Engagement was defined as participants completing at least one module in the WebMAP program. They found that 14 of 15 (93%) participants met this threshold, which was higher than the proportion of youth (40/57, 70%) who engaged with any iCanCope content. Second, Phillips et al reported engagement with an app-based program called Voice Crisis Alert V2 over a 12-week intervention period [35]. They characterized engagement based on the use of individual app components, similar to the content categorization approach used in our study. Among 60 dyads, symptom tracking and history were the most popular features (used by 90% of dyads), while a clinician messaging feature was least popular (total of 5 message threads generated during the study). In our study, we similarly found that symptom tracking and history were among the most popular content areas. Third, Saulsberry et al offered the Sickle Cell Transition E-Learning Program to 183 youth, of whom, 53 (29%) completed at least one of the 6 available modules [36]. In our study, a higher proportion of youth (70%) who were offered the iCanCope program engaged with the content. Fourth, Leonard et al reported engagement with a medication adherence app as percentage of days that each participant logged medication administration [34]. In a pilot sample of 11 patients, average engagement was 80%. Our study found a wide range of youth engagement with various program components, with the most popular feature being goal setting (35/40, 88%) and the least popular being education about SCD and treatment (6/40, 15%).

The iCanCope program has also been assessed in youth with juvenile idiopathic arthritis and chronic pain [24,25]. Usage levels were similar in the SCD population, although engagement was higher with the symptom history function in the juvenile idiopathic arthritis population [25] at 80% versus 60% among SCD users. A systematic review focusing on engagement and adherence to mHealth interventions in children and youth in clinical and nonclinical populations reported average adherence of 78% [37].

Several contextual factors may explain the differences in engagement patterns seen across studies in the SCD population, among users of other iterations of the iCanCope program, and generally among the pediatric population. These factors include differences in the intervention scope (eg, pain management versus transition readiness), distinct definitions of engagement, different study designs and clinical populations, and technology variation (eg, wearable technology vs app based) [38]. The literature suggests that treatment adherence among youth with SCD may be impacted by forgetfulness, side effects of pharmacological interventions (eg, nausea, heart racing, and taste aversion), questioning efficacy of treatment, and a desire to be “normal,” and some of these factors may similarly impact adherence to digital health interventions [39]. Previous literature also suggests that sociodemographic factors can impact engagement with digital interventions, highlighting lower rates of engagement among racial or ethnic minorities, and those with low socioeconomic status [40-42].

There is limited research on caregiver engagement with digital health interventions for SCD. Many interventions designed for youth do not have a caregiver component. However, 3 recent digital health studies have reported parent engagement in pediatric SCD, chronic pain, and cancer populations, respectively [43,44]. The WebMAP program was designed for pediatric chronic pain patients and adapted for the SCD population. In the chronic pain population, caregiver engagement was higher compared with youth (82% vs 74% completed at least one module) [43]. Similarly, caregiver engagement was also higher (100% vs 93% completed at least one module) than youth engagement in the SCD population [27]. In contrast, the Teens Taking Charge Cancer web-based self-management program [44] reported lower caregiver engagement compared with youth (8% vs 28% completed at least one module), which is consistent with our results. One study on the psychology of eHealth use among caregivers with children having complex health conditions found that factors, such as poor caregiver psychosocial health, high eHealth literacy, and high acceptability of eHealth, were associated with increased engagement [45]. Furthermore, caregiver engagement with nondigital or analog health interventions that report high levels of engagement tend to include methods, such as motivational interviewing and teaching-learning processes (eg, role play and interactive problem solving) [46]. Our program was self-guided, which may have contributed to lower levels of engagement from caregivers.

### Strengths and Limitations

Our study had several strengths. First, a phased evidence-based approach was used to develop the iCanCope with SCD program. A qualitative needs assessment involving youth with SCD, caregivers, and health care providers was completed to determine essential components of the program [12]. Next, design sessions were completed with patient partners with SCD to adapt the platform and content for a population with SCD [26]. The changes that were implemented (eg, content tailoring and app design) were cited by participants and their caregivers as positive aspects of the iCanCope program. Second, this study used both qualitative and quantitative methods, which enhanced our ability to understand and contextualize the diverse set of engagement experiences. Furthermore, by including youth and caregiver perspectives, we were able to distinguish their unique experiences and understand how the family unit engaged with the program. Third, this study was a multi-center randomized controlled trial, and this increases the generalizability of the findings to SCD patients in North America. Moreover, the study sample was inclusive of families from a variety of socioeconomic backgrounds.

These findings should be considered in the context of a few limitations. First, caregivers were not heavily included in the design of the iCanCope program, which may partially explain their reduced engagement with the content. As per the user design approach, the creation of iCanCope was focused on meeting the needs of youth, as the primary users were envisioned to be youth, rather than caregivers. However, our study findings indicate that parent engagement with SCD program content is important as it is positively associated with child engagement. Unfortunately, poststudy interviews with caregivers revealed that the program was too content heavy and time-consuming. One strategy to improve caregiver engagement would be to account for the individual need for cognition, which refers to the enjoyment of deep thinking [47]. For those with a low need for cognition, information could be provided in short easily accessible excerpts, such as videos or infographics. In contrast, long-form content would be more suited for individuals with a high need for cognition. Notably, once the program is publicly released, caregivers will be able to review content at their own pace rather than within the time constraints of a research study. In addition, consultations with parents may be completed to fine tune the program to meet their needs prior to public release. Second, differential use of the app versus website among youth suggests a need for changes to the website to increase engagement with the overall program. A specific technical limitation of the website was that it was unable to store the responses of participants, making it difficult for them to gauge their progress. Finally, we were only able to interview 1 youth participant who did not engage with the program at all.

### Future Directions

Prior to public release of the program, there are opportunities for refinement using the elements of persuasive design [48]. Wen et al found that more frequent prompts were associated with higher engagement in studies with clinical pediatric populations [37]. Although the iCanCope app did incorporate reminders to prompt participants to engage (eg, push notifications to complete check-ins or set a goal), the frequency and timing of the reminders could be customized. However, it is important to balance the number of reminders while minimizing interruptions to the user. As such, identifying ideal opportunities to use the iCanCope program individualized to each participant is important. A novel approach includes using algorithms to determine these opportunities, which have been developed for adult populations [45,49]. In tandem, providing praise (eg, congratulatory messaging) and rewards when participants complete a check-in, read an article from the library, or complete a goal may influence engagement positively [48]. Rewards can include elements of gamification where participants gain points or virtual rewards for completing certain tasks, which they can use to promote engagement with self-management content. Finally, real-world use of the iCanCope program can also be studied in the future, after public release.

In terms of program dissemination, using strategies to target the family unit, rather than individual members, may increase engagement as SCD typically impacts multiple members of a family. This approach is supported by youth participants who reported *ad hoc* sharing of program content with their siblings and the finding that parent and child engagement was positively associated. Furthermore, by partnering with SCD clinics, the program could be introduced to youth and families when the SCD pain becomes more frequent to help cope with the corresponding increase in pain burden.

### Conclusions

This is one of the first studies to apply digital health analytics to characterize patterns of engagement with SCD self-management among youth and caregivers. Differential engagement patterns were found, with more youth engaging in the program than their caregivers; however, youth were more likely to engage with the app than the website. These findings will be used to optimize the iCanCope with SCD program prior to release, with the potential to improve the accessibility and acceptability of pain self-management support for families affected by SCD.

## Appendix

Multimedia Appendix 1 iCanCope with sickle cell disease program screenshots.

## Abbreviations

SCD: sickle cell disease
WebMAP: web-based management of adolescent pain

## Data Availability

The data sets generated during and analyzed during this study are available from the corresponding author on reasonable request.
