# Supplementary material for: Characterizing User Engagement With a Digital Intervention for Pain Self-management Among Youth With Sickle Cell Disease and Their Caregivers: Subanalysis of a Randomized Controlled Trial
Source: J Med Internet Res. 2022 Aug 30;24(8):e40096. doi: 10.2196/40096 (PMC9472047; doi:10.2196/40096)
Supplement: Multimedia Appendix 1 [file jmir_v24i8e40096_app1.docx]

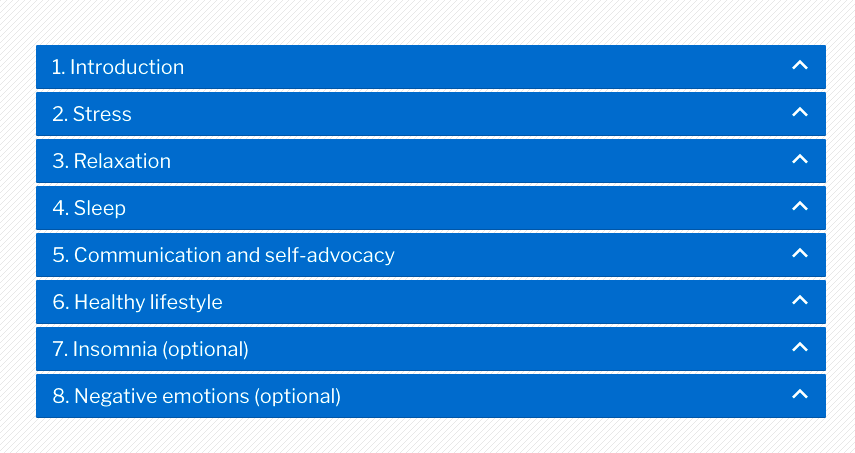


Figure S1. iCanCope with Sickle Cell Disease website navigation flow for youth participants.


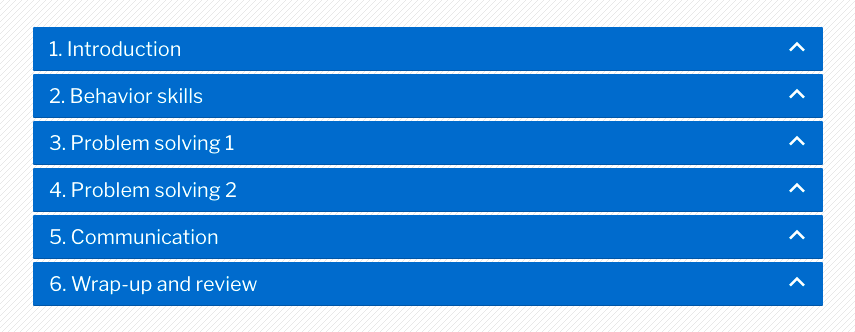


Figure S2. iCanCope with Sickle Cell Disease website navigation flow for caregiver participants.


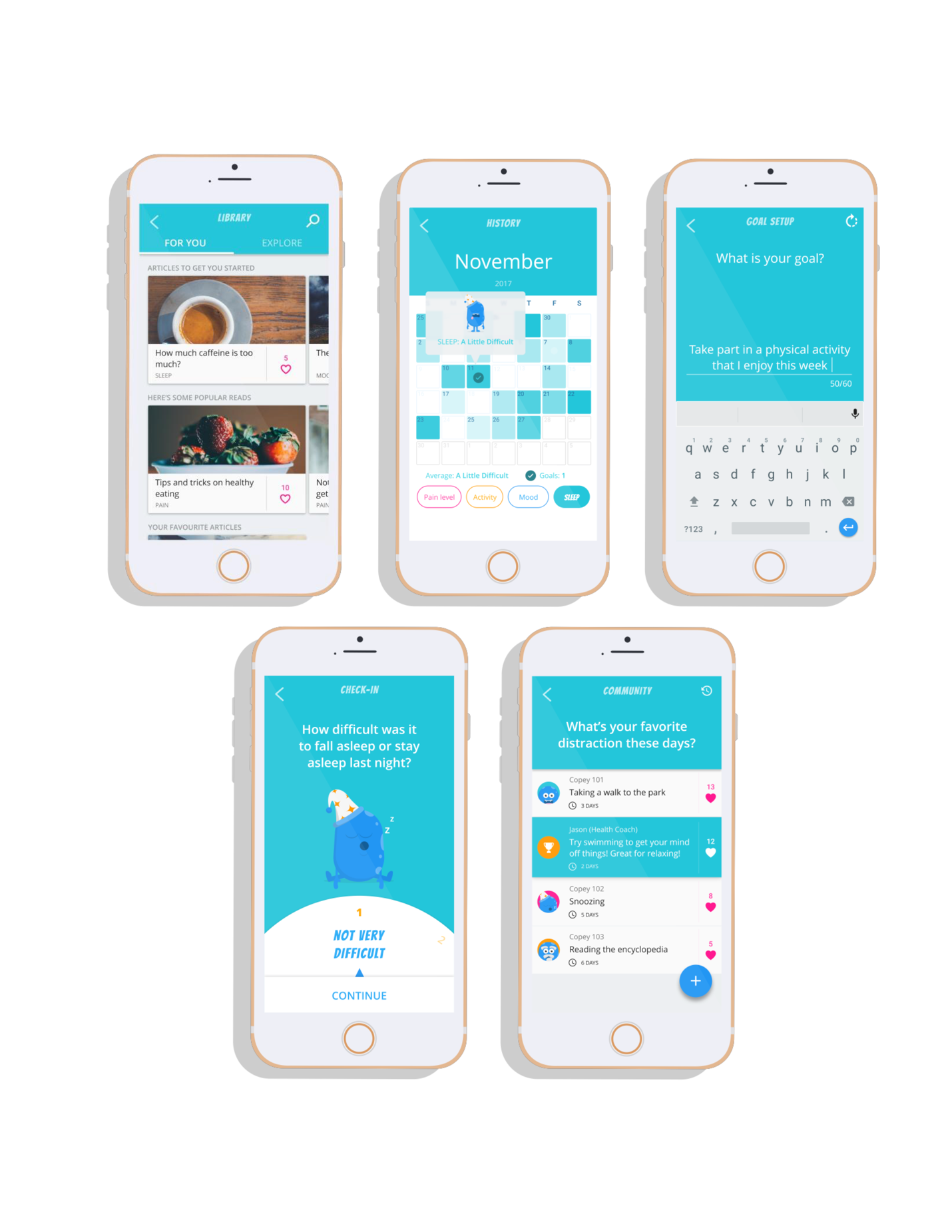


Figure S3. Screenshots of the iCanCope with Sickle Cell Disease app.
